# Brain Microenvironment Heterogeneity: Potential Value for Brain Tumors

**DOI:** 10.3389/fonc.2021.714428

**Published:** 2021-09-01

**Authors:** Laura Álvaro-Espinosa, Ana de Pablos-Aragoneses, Manuel Valiente, Neibla Priego

**Affiliations:** Brain Metastasis Group, Molecular Oncology Programme, Spanish National Cancer Research Centre (CNIO), Madrid, Spain

**Keywords:** brain, brain metastasis, microenvironment, heterogeneity, single-cell analysis

## Abstract

Uncovering the complexity of the microenvironment that emerges in brain disorders is key to identify potential vulnerabilities that might help challenging diseases affecting this organ. Recently, genomic and proteomic analyses, especially at the single cell level, have reported previously unrecognized diversity within brain cell types. The complexity of the brain microenvironment increases during disease partly due to the immune infiltration from the periphery that contributes to redefine the brain connectome by establishing a new crosstalk with resident brain cell types. Within the rewired brain ecosystem, glial cell subpopulations are emerging hubs modulating the dialogue between the Immune System and the Central Nervous System with important consequences in the progression of brain tumors and other disorders. Single cell technologies are crucial not only to define and track the origin of disease-associated cell types, but also to identify their molecular similarities and differences that might be linked to specific brain injuries. These altered molecular patterns derived from reprogramming the healthy brain into an injured organ, might provide a new generation of therapeutic targets to challenge highly prevalent and lethal brain disorders that remain incurable with unprecedented specificity and limited toxicities. In this perspective, we present the most relevant clinical and pre-clinical work regarding the characterization of the heterogeneity within different components of the microenvironment in the healthy and injured brain with a special interest on single cell analysis. Finally, we discuss how understanding the diversity of the brain microenvironment could be exploited for translational purposes, particularly in primary and secondary tumors affecting the brain.

## Introduction

The brain microenvironment represents a complex habitat that notably differs from the microenvironment associated with other tumors (1). In addition to the still incomplete understanding of brain homeostasis and the structural heterogeneity of this organ, the presence of any insult, such as a tumor, might contribute to amplify the pre-existing diversity within the microenvironment.

Imaging, genomic and proteomic analyses have been valuable tools for dissecting inter- and intra-regional heterogeneity within the brain. Initially applied to uncover neuronal subtypes across brain regions (2–5), single-cell RNA sequencing (scRNAseq), single-nucleus RNA sequencing (snRNAseq), mass cytometry (CyTOF) and spatial transcriptomics, have also proved to be a powerful tool beyond non-neuronal cells, revolutionizing the way we interrogate cancer-associated heterogeneity. Recently, the principles of scRNAseq have been expanded to elucidate *in vivo* networks based on cell-to-single cell interactions (6–8). These studies are dramatically expanding the complexity of the brain that should be translated into comprehensive pharmacologic approaches overcoming initial technical difficulties associated with this organ (9).

**Figure 1:**
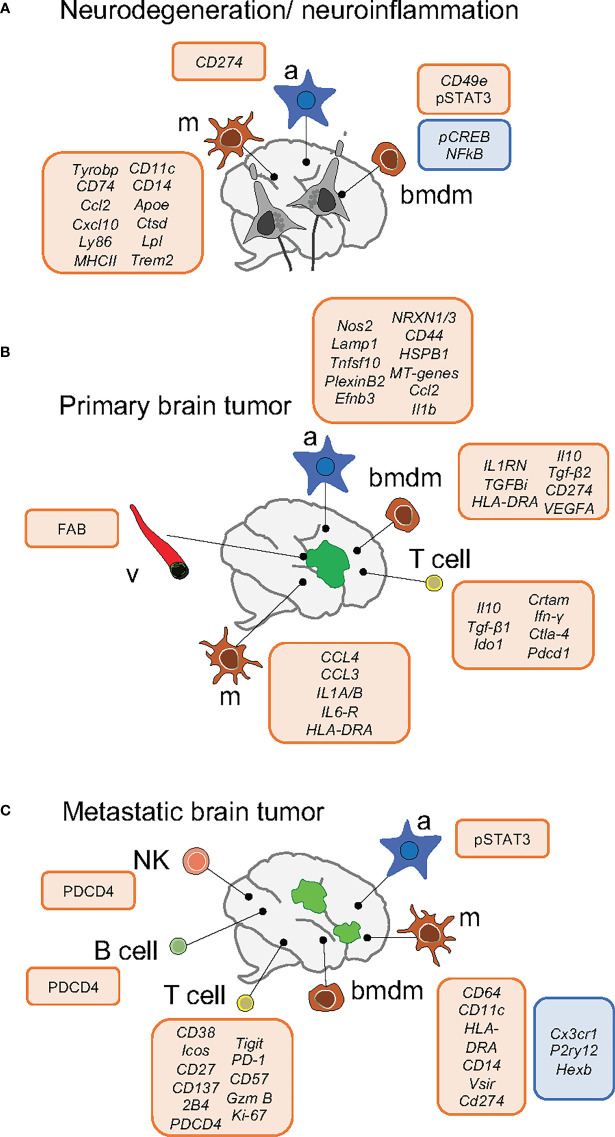
Schema of key markers deregulated within the brain microenvironment in neurodegeneration/neuroinflammation **(A)** and primary **(B)** and secondary **(C)** brain tumors. Upregulation is indicated by the box in red and downregulation by the box in blue. Neurodegeneration/neuroinflammation comprises the following brain disorders: Alzheimer, Huntington disease, Amyotrophic Lateral Sclerosis and Experimental Autoimmune Encephalomyelitis. a, astrocytes; m, microglia; bmdm, bone marrow-derived macrophages; v, vasculature.

Although the characterization of altered molecular pathways within the brain microenvironment at the single cell level in brain tumors, especially in brain metastasis, is still limited, in this perspective we take advantage of findings obtained from other contexts (**Figure 1**) to discuss how exploiting heterogeneity could be translated into novel therapeutic strategies also for brain tumors.

## Diversity of Macrophages Within the Brain Microenvironment

### Health and Aging

scRNA-seq approaches have uncovered specific transcriptomic profiles that distinguish brain microglia and macrophages (2, 10–13). Additionally, different microglial states have been found at embryonic and early postnatal time points (14–16), while aging modulates inflammatory and interferon response signatures in microglia (14), as detailed in **Table 1**.

**Table 1:** Key signatures and markers found in microglia/macrophages, T cells, astrocytes and endothelial cells subpopulations within the brain in preclinical models and/or patients of brain disorders and primary and secondary brain tumors.

Paper	PMID	Context	Cell type	Gene	Up/Down	Validated in patients	Notes
Masuda et al.	30760929	Health	Microglia	*TMEM119, P2RY12, CX3CR1, P2RY13, SLC2A5*	Defining signature	Yes (Human samples)	
Zeisel et al.	25700174	Health	Microglia	*Aif1 (Iba1), Cx3cr1*	Defining signature	No	
Zeisel et al.	25700174	Health	pvMΦ	*Aif1 (Iba-1), Cx3CR1, Mrc1 (CD206), Lyve1, Lyl1, Spic*	Defining signature	No	
Goldmann et al.	27135602	Health	pvMΦ	*Aif1 (Iba-1), Cx3CR1, Csf1r, CD45 (Ptprc)high, Mrc1, CD36*	Defining signature	No	
Goldmann et al.	27135602	Health	Microglia	*Aif1 (Iba-1), Cx3CR1, Csf1r, CD45low, P2ry12*	Defining signature	No	
Jordao et al.	30679343	Health	Microglia	*P2ry12, Tmem119, Sparc, Olfml3, Sall1*	Defining signature	No	
Jordao et al.	30679343	Health	BAMs/CAMs	*Mrc1, Pf4, Ms4a7, Cbr2*	Defining signature	No	
Jordao et al.	30679343	Health	mMΦ	*Mrc1, Pf4,Ms4a7, Stab1, Cbr2, Cd163, Fcrl, Siglec1*	Defining signature	No	
Van Hove et al.	31061494	Health	BAMs/CAMs	*Apoe, Ms4a7, Ms4a6c, Lyz2, Tgfbi*	Defining signature	No	
Li et al.	30606613	Health	Microglia	*Tmem119, P2ry12*	Defining signature	No	
Mrdjen et al.	29426702	Aging	Microglia	*CD11c, CD14* (phagocytosis markers), *CD44, CD86, PD-L1*	Up	No	
Mrdjen et al.	29426702	Aging	Microglia	*CX3CR1, MerTK, and Siglec-H* (core microglia genes)	Down	No	
Hammond et al.	30471926	Aging	Microglia	*OA2: Lgals3, Cst7, Ccl3, Ccl4, Il1b* (pro-inflammatory) *2) OA3: Ifitm3, Trp4, Oasl2* (IFN-response genes)	Up	No	
Keren-Shaul et al.	28602351	Alzheimer	Microglia (DAM)	*Apoe, Ctsd, Lpl, Tyrobp, Trem2, CD11c (Itgax)*	Up	No	
Keren-Shaul et al.	28602351	Alzheimer	Microglia (DAM)	*P2ry12/P2ry13, Cx3cr1, Tmem119* (core microglia genes)	Down	No	
Mathys et al.	29020624	Alzheimer	Microglia (late-response)	*Apoe, Axl, Lgals3bp + H2-Ab1, H2-D1, CD74* (antigen presentation-related genes)	Up	No	
Mathys et al.	29020624	Alzheimer	Microglia	*Cx3cR1, P2ry12, TMEM119* (core microglia genes)	Down	No	
Mrdjen et al.	29426702	Alzheimer	Microglia	*CD11c, CD14* (phagocytosis markers)*, CD44, CD86, PD-L1*	Up	No	
Mrdjen et al.	29426702	Alzheimer	Microglia	*CX3CR1, MerTK, and Siglec-H* (core microglia genes)	Down	No	
Habib et al.	32341542	Alzheimer	Microglia	*Apoe, Ctsd, Ctsb, Ctsl*	Up	No	
Mathys et al.	31042697	Alzheimer	Microglia (DAM)	*Apoe, Trem2, CD74, Hla-drb1/5*	Up	Yes	
Olah et al.	33257666	Alzheimer	Microglia	*CD74, ISG15, CD83*	Up	Yes	Several microglia clusters, each one characterized (and validated) with these genes
Keren-Shaul et al.	28602351	Amyotrophic Lateral Sclerosis	Microglia	*Tmem119, P2ry12 (core microglia genes)*	Down	No	
Masuda et al.	30760929	Multiple sclerosis	Microglia	*TMEM119, P2RY12, P2RY13, CX3CR1, SLC2A5* (core microglia genes)	Down	Yes	
Masuda et al.	30760929	Multiple sclerosis	Microglia	*APOE, MAFB*	Up	Yes	
Mrdjen et al.	29426702	Multiple sclerosis (EAE)	Microglia	*CX3CR1, MerTK* and *Siglec-H* (core microglia genes)	Down	No	
Mrdjen et al.	29426702	Multiple sclerosis (EAE)	Microglia	*MHCII, Sca-1, PDL1, CD11c, CD44, CD86*	Up	No	
Mrdjen et al.	29426702	Multiple sclerosis (EAE)	Microglia	*CD14*	Down	No	
Jordao et al.	30679343	Multiple sclerosis (EAE)	Microglia	*P2ry12, Tmem119, Selplg, Siglech, Gpr34, Sall1* (core microglia genes)	Down	No	
Jordao et al.	30679343	Multiple sclerosis (EAE)	Microglia	*Ly86, CCl2, Cxcl10, Mki67*	Up	No	
Jordao et al.	30679343	Multiple sclerosis (EAE)	Microglia	*Sparc, Olfml3*	Up	No	
Jordao et al.	30679343	Multiple sclerosis (EAE)	Microglia	*1) damicroglia2: Cd74, Ctsb, Apoe 2) damicroglia3: Cxcl10, Tnf, Ccl4 3) damicroglia4: Ccl5, Ctss, Itm2b*	Up	No	3 clusters
Jordao et al.	30679343	Multiple sclerosis (EAE)	BMDM	*Mertk, Mrc1, Zbtb46, Cd209a*	Up	No	
Ajami et al.	29507414	Multiple sclerosis (EAE)	CNS-resident myeloid cells (microglia, pvMΦ, mMΦ)	*MHCII, CD86, CD80, Axl, Tim4, CD274 (Pd-l1), CD195 (Ccr5), CD194 (Ccr4), CD11c (Itgax)*	Up	No	
Ajami et al.	29507414	Multiple sclerosis (EAE)	BMDM	*CD80, CD86, CD38, CD39, MerTK, Axl, CD206, TREM2, CD274*	Up	No	
Ajami et al.	29507414	Multiple sclerosis (EAE)	BMDM	pSTAT3	Up	No	
Ajami et al.	29507414	Multiple sclerosis (EAE)	BMDM	pCREB*, NFkB*	Down	No	
Ajami et al.	29507414	Multiple sclerosis (EAE)	BMDM	*CD49e (itga5)*	Up	No	
Hammond et al.	30471926	Multiple sclerosis (LPC-induced demyelination)	Microglia	*P2ry12, Cx3cr1 (core microglia genes)*	Down	No	
Hammond et al.	30471926	Multiple sclerosis (LPC-induced demyelination)	Microglia	*CxCl10, Ccl4, Ifi204, Apoe, Lpl, Spp1*	Up	No	
Rothhammer et al.	29769726	Multiple sclerosis (EAE)	Microglia	*AHR*	Up	Yes	Functional validation
Rothhammer et al.	29769726	Multiple sclerosis (EAE)	Microglia	*TGF-alpha*	Up	Yes	Functional validation
Rothhammer et al.	29769726	Multiple sclerosis (EAE)	Microglia	*VEGF-B*	Up	Yes	Functional validation
Clark et al.	33888612	Multiple sclerosis (EAE)	Microglia	*Semad4d*	Expressed in EAE	Yes	Functional validation
Clark et al.	33888612	Multiple sclerosis (EAE)	Microglia	*Ephb3*	Expressed in EAE	Yes	Functional validation
Friebel et al.	32470397	Glioma	Microglia and BMDM	*CD64, CD11c, HLA-DR, CD14*	Up	Yes	
Friebel et al.	32470397	Glioma	BMDM	*CD45RA, CD141, Icam*	Up	Yes	
Friebel et al.	32470397	Glioma	BMDM	*CD38, PD-L1, PD-L2*	Up	Yes	
Darmanis et al.	29091775	Glioma	Microglia	*CCL3, CCL4, CCL2,TNF (*Cks)*. IL1A/B, IL6-R* (pro-inflammatory)	Up	Yes	
Darmanis et al.	29091775	Glioma	BMDM	*VEGFA, VEGFB* (angiogenesis)*, IL1RN, TGFBi* (anti-inflammatory)	Up	Yes	
Ochocka et al.	33809675	Glioma	Microglia and BMDM	*H2-Aa, H2-Ab1, H2-D1, H2K1(MHCII), Ifitm3*	Up	No	
Ochocka et al.	33809675	Glioma	Microglia	*Ccl3, Ccl4, Ccl12*	Up	No	
Ochocka et al.	33809675	Glioma	BMDM	*Cd274, il1rn, il18b*,	Up	No	
Sankowski et al.	31740814	Glioma	Microglia	*CX3CR1, CSF1R*	Down	Yes	
Sankowski et al.	31740814	Glioma	Microglia	*CD163, APOE, SPP1, TREM2 LPL, IFI27, IFITM3, HIF1A, VEGFA*	Up	Yes	
Friebel et al.	32470397	Brain Metastasis	CNS resident (microglia) and BMDM	*CD64, CD11c, HLA-DR, CD14*	Up	Yes	
Friebel et al.	32470397	Brain Metastasis	BMDM	*CD45RA, CD141, ICAM*	Up	Yes	
Friebel et al.	32470397	Brain Metastasis	BMDM	*CD38, PD-L1, PD-L2*	Up	Yes	
Guldner et al.	33113353	Brain Metastasis	CNS-myeloids (microglia+BAMs)	*S100a11, Lgals, Il1b*	Up	No	
Guldner et al.	33113353	Brain Metastasis	CNS-myeloids (microglia+BAMs)	*Cx3cr1, P2ry12, Hexb* (core microglia genes)	Down	No	
Guldner et al.	33113353	Brain Metastasis	CNS-myeloids (microglia+BAMs)	*Vsir, Cd274*	Up	No	
Guldner et al.	33113353	Brain Metastasis	BMDM	*Tspo, Isg15, Ifitm2, Anxa2, Irf7* (inflammation)*,Ifitm1, Il1b, S100a10, Lgals1*	Up	No	
Guldner et al.	33113353	Brain Metastasis	BMDM	*Hbb-bs, Serinc3, CD81, Klf2*	Down	No	
Korin et al.	28758994	Health	Brain infiltrated leukocytes	*CXCR1*	Up (compared with blood)	No	
Korin et al.	28758994	Health	Brain infiltrated leukocytes	*CD44*	Up (compared with blood)	No	
Korin et al.	28758994	Health	CD8+ T cells	*CD86*	Up (compared with blood)	No	
Golomb et al.	33264626	Ageing	CD4+/CD8+ T cells	T memory stemness (Tscm) signature: CD3+ *and Thy1+/Itga2+/Klrb1-* mRNA	Up (compared with young mice)	No	
Caruso et al.	33155039	Glioma	CD8+ T cells	*CRTAM*	Up	Patients data	
Friebel et al.	32470397	Brain Metastasis	CD8+ T cells	*CD38*	Up (compared with the cluster of low immune infiltrates and worse survival)	Patients data	
Friebel et al.	32470397	Brain Metastasis	CD8+ T cells	Co-stimulatory receptors: *Icos, CD27 and CD137*	Up (compared with the cluster of low immune infiltrates and worse survival)	Patients data	
Friebel et al.	32470397	Brain Metastasis	CD8+ T cells	Co-inhibitory receptors: *2B4, Tigit and Pd-1*	Up (compared with the cluster of low immune infiltrates and worse survival)	Patients data	
Friebel et al.	32470397	Brain Metastasis	CD8+ T cells	Effector function: *CD57 a*nd *gzmB*	Up (compared with the cluster of low immune infiltrates and worse survival)	Patients data	
Friebel et al.	32470397	Brain Metastasis	CD8+ T cells	*Ki-67*	Up (compared with the cluster of low immune infiltrates and worse survival)	Patients data	
Boisvert et al.	29298427	Aging	Astrocytes	*Casp1*	Up	No	
Boisvert et al.	29298427	Aging	Astrocytes	*Casp12*	Up	No	
Boisvert et al.	29298427	Aging	Astrocytes	*Cxcl5*	Up	No	
Boisvert et al.	29298427	Aging	Astrocytes	*Tlr2*	Up	No	
Boisvert et al.	29298427	Aging	Astrocytes	*Tlr4*	Up	No	
Lau et al.	32989152	Alzheimer	Astrocytes	*ADGRV1*	Defining signature	Yes	
Lau et al.	32989152	Alzheimer	Astrocytes	*GPC5*	Defining signature	Yes	
Lau et al.	32989152	Alzheimer	Astrocytes	*RYR3*	Novel gene signature identifying astrocytes	Yes	
Lau et al.	32989152	Alzheimer	Astrocytes	*NRXN1*	Down	Yes	
Lau et al.	32989152	Alzheimer	Astrocytes	*NRXN3*	Down	Yes	
Leng et al.	33432193	Alzheimer	Astrocytes	*HSPB1*	Up	Yes	Expression validated in a mouse model of spinal cord injury
Leng et al.	33432193	Alzheimer	Astrocytes	*TNC*	Up	Yes	Expression validated in a mouse model of spinal cord injury
Leng et al.	33432193	Alzheimer	Astrocytes	*HSP90AA1*	Up	Yes	Expression validated in a mouse model of spinal cord injury
Leng et al.	33432193	Alzheimer	Astrocytes	Glutamate/GABA-signalling associated genes	Down	Yes	Expression validated in a mouse model of spinal cord injury
Al-Dalahmah et al.	32070434	Huntington disease	Astrocytes	*MT-genes*	Up	Yes	
Al-Dalahmah et al.	32070434	Huntington disease	Astrocytes	Protoplasmic genes	Down	Yes	
Rothhammer et al.	29769726	Multiple sclerosis (EAE)	Astrocytes	*Ccl2*	Upregulated upon AHR deletion	No	Functional validation
Rothhammer et al.	29769726	Multiple sclerosis (EAE)	Astrocytes	*Il1b*	Upregulated upon AHR deletion	No	
Rothhammer et al.	29769726	Multiple sclerosis (EAE)	Astrocytes	*Nos2*	Upregulated upon AHR deletion	No	
Sanmarco et al.	33408417	Multiple sclerosis (EAE)	Astrocytes	*CD107a (Lamp1)*	Upregulated upon CNS inflammation	Yes	Functional validation
Sanmarco et al.	33408417	Multiple sclerosis (EAE)	Astrocytes	*Tnfsf10* (TRAIL death receptor ligand)	Upregulated upon CNS inflammation	Yes	Functional validation
Clark et al.	33888612	Multiple sclerosis (EAE)	Astrocytes	*PlexinB2*	Expressed in EAE	Yes	Functional validation
Clark et al.	33888612	Multiple sclerosis (EAE)	Astrocytes	*Efnb3*	Expressed in EAE	Yes	Functional validation
Heiland et al.	31186414	Glioblastoma	Reactive astrocytes	*CD274*	Up	Yes (human samples)	
Priego et al.	29921958	Brain Metastasis	Reactive astrocytes	STAT3 (phosphorilation)	Up	Yes	Functional validation
Ebert et al.	33082953	Glioblastoma	Pericytes	CD73 CD105	Up	Yes (human samples)	
Ebert et al.	33082953	Glioblastoma	Tumor associated endothelial cells	Fab	Up	Yes (human samples)	
Carlson et al.	33367832	Glioblastoma	Tumor associated endothelial cells	*Jcad, Spop* and *Ctnnb1* (in all clusters), clusters 2–5: *Malat1, Jun* and *Arhgap*, cluster 3: *Mgp, Stmn2, Sema3g* and *Gja4*, cluster 4: *Nr2f2, Vwf, Aldh1a1* and *Junb*, cluster 5: *CD74 a*nd *Cxcl10*	Up	No	Validation in patient derived orthotopic xenograft
Carlson et al.	33367832	Glioblastoma	Tumor derived endothelial cells	*Pdpn* and *Flt4* Lymphatic endothelial cells: *Icam1, Dcn, Tgfbi and CD74*	Up	No	Validation in patient derived orthotopic xenograft

BAM, Barrier-associated macrophages; CAM, Central Nervous System (CNS)-associated macrophages; BMDM, Bone Marrow-Derived Macrophages; DAM, Disease-associated microglia; mMφ, meningeal macrophages; pvMφ, perivascular macrophages.

### Brain Disorders

During Alzheimer disease (AD), disease-associated microglia (DAM) and late-response microglia are defined by the expression of genes related to lipid metabolism and phagocytosis (*ApoE, Lpl, Trem2, Tyrobp, Ctsd*) and interferon response (17, 18). By combining CyTOF with lineage tracing models Mrdjen et al. identified a subset of microglia during AD characterized by the upregulation of phagocytic markers CD11c and CD14. However, the specific functional contribution of DAMs during AD remains unclear (17, 19). During Experimental Autoimmune Encephalomyelitis (EAE) microglia showed a similar signature, except for decreased CD14 and increased MHCII and Sca-1 expression (11). In the same line, Ajami et al. identified two CNS-resident myeloid populations increased in frequency during EAE, Amyotrophic Lateral Sclerosis (ALS) and Huntington’s disease (HD) (20) and Jordao et al. described four disease-associated microglia in EAE (**Table 1** details defining gene signatures). Peripheral monocyte populations present in the EAE model, but absent in AD and HD, express CD49e and show higher expression of pSTAT3 and lower of pCREB and NFκ-B in comparison to resident myeloid cells.

Remarkably, high-throughput technological pipelines are now available to profile novel cell-to-cell interactions at a single cell level. Clark et al. combines molecular barcoding, viral tracing and scRNASeq *in vivo* (RABID-seq) to map the microglia-astrocyte crosstalk during EAE, being responsible of inducing a pro-inflammatory microenvironment through two main axes: *Sema4D-PlexinB2* and *Ephrin-B3/EphB3* (8).

As summarized in **Table 1**, analysis of human and mouse microglia suggests high correlation in their transcriptomic profiles (i.e. upregulation of *Apoe)* and highlight the broader heterogeneity of human microglia (15, 21–23).

### Brain Tumors

Recent sc-RNAseq analysis found that the interaction of tumor-associated macrophages (TAMs) and glioma cells occurs mainly through CXCL chemokines and their receptors (24). Furthermore, scRNA-seq analysis of CD11b+ myeloid cells isolated from murine experimental GL261 gliomas unveiled that activated microglia and BMDM significantly change their transcriptional networks, with upregulation of MHCII related proteins (25). In glioma patients, TAM BMDM invade the tumor core displaying an anti-inflammatory and pro-angiogenic phenotype, expressing immunosuppressive cytokines (i.e. *Il10* and *Tgfβ2*) and markers of active phagocytosis (*CD93*). Meanwhile, microglia located in the surrounding space is characterized by the expression of pro-inflammatory molecules (i.e. C*CL4, CCL3, IL1A/B*) (25–27).

Recently this heterogeneity has also been addressed in brain metastasis in comparison to gliomas. Friebel et al. found that, while the glioma microenvironment is predominantly composed by activated microglia, brain metastases are characterized by the infiltration of BMDM (28). Similarly, Guldner et al. identified myeloid clusters characterized by the expression of complement genes, while BMDMs express higher levels of inflammatory genes (*S100a11, Lgals, Il1b*) in brain metastasis. Furthermore, it was shown that loss of *Cx3cr1* in CNS-myeloid cells triggers upregulation of *Cxcl10*, which in turn drives an immunosuppressive pro-metastatic microenvironment through PD-L1 and VISTA. Interestingly, co-inhibition of both molecules reduced the brain metastatic burden (29).

## Lymphocytes and Natural Killer Cells Heterogeneity Within the Brain Microenvironment

### Health and Ageing

Applying CyTOF to the naïve mouse brain, Korine et al. found that CD4+ and CD8+ infiltrating T cells express markers of memory T cells (CD44+CD62L-) and could be characterized by the increased expression of CD86 and CX3CR1 in comparison to their blood counterparts. Indeed, CD44 was suggested to be a general marker for brain infiltrating immune populations (30). Brain B cells and NK cells, which are found in lower numbers than in peripheral blood, particularly IgM+ B cells, are also defined by CX3CR1 expression (30). In the aged brain, using cellular indexing of transcriptomes and epitopes by sequencing (CITE-seq), T cells were found to express a T cell memory stemness signature characterize by CD3+ and Thy1+/Itga2+/Klrb1- mRNA expression and additional gene signatures associated with chemotaxis and ribosomal proteins, including Ly6a and Dusp2 expression. These findings suggest that organismal aging correlates with the enrichment of specific lymphocytes populations within the brain (31).

### Brain Tumors

Single-cell transcriptomics uncovered a gene signature in glioma composed by immune effector molecules and inhibitory feedback mechanisms (genes such as *Ifn-γ, Ctla-4, Pdcd1, IL-10, Tgf-β1 or Ido1*) that lead to the reprogramming of T cells subsets that become unable to target the cancer cells (32). A more specific dissection of the crosstalk between glioma cells and T cells in patients was achieved by applying single-cell Tumor-Host Interaction (scTHI) analysis of scRNA sequencing data. In particular, Caruso et al. found that the cross-talk between CD8+ T cells and tumor cells included components belonging to major histocompatibility complex Class I, chemokines, interleukins, IFN-γ and TNF. This study also described paracrine interactions with myeloid cells involving immune checkpoint genes, TNF family members and chemoattractant chemokine ligands, such as CXCR6 receptor on T cells and its ligand CXCL16 secreted by macrophages that are upregulated in glioma (7).

Cy-TOF of surgical resections have characterized the lymphocyte landscape in primary and secondary brain tumor entities (28).Compared to primary brain tumors, metastases favor T and B cell infiltration and T regulatory cells (T regs) present higher accumulation in brain metastasis and IDH1 *wt* gliomas. Moreover, CD8+ T cells present an increased expression of co-stimulatory and co-inhibitory receptors, the activation marker CD38 and effector and proliferation functions in metastases, while glioma samples show less activation (28). The activation/exhaustion phenotypic state of T cells in metastatic tumors could explain their favorable clinical response to immune checkpoint inhibitors compared to those of primary origin.

Recent papers shed light on the stromal and immune landscape in human multiple sclerosis and brain tumors, focusing on the analysis by scRNAseq and set enrichment analysis of cerebrospinal fluid (CSF) leukocytes (33), and CSF from patients (34, 35). Specifically, Rubio-Perez et al. have described an inflammatory status independently of the primary tumor source of the metastasis and a cluster characterized by active proliferation of T cells. Noteworthy, identical T cell receptor sequences between the CSF and the metastatic lesions were detected in 66.7% of patients, indicating a partial connection of the immune profiles from both compartments (34). This work suggests the potential value of CSF to characterize the immune microenvironment and T cells subclonal evolution in brain metastasis to monitor patients during tumor progression or treatment.

## Astrocytes Diversity Within the Brain Microenvironment

### Health and Aging

Different studies have shown that astrocytic transcriptome heterogeneity encompasses well-recognized astrocyte functions and happens both between and within brain regions (9, 36). In aged brains, cerebellar astrocytes were characterized by the upregulation of inflammatory factors that can damage synapses (*caspase-1* and -*12*, Cxcl5) and key inflammasome receptors *Tlr2* and *4*. This demonstrates that dependency of the glial cell type correlates with more severe or less synaptic dysfunction (37).

### Brain Disorders

Astrocytes can be rapidly activated in response to various insults, by a process known as “reactive astrogliosis” which aims to limit the damage that occurs locally. Three states of reactive astrocytes (RAs) can be found in HD, defined by different levels of GFAP, metallothionein (MT) genes and quiescent protoplasmic genes. The upregulation of MTs by RAs could be a protective response to combat oxidative stress, which is characteristic of the HD brain (38). Interestingly, astrocytes in AD were found to express a unique and novel signature (*Adgrv1*, *Gpc5* and *Ryr3 genes*). Down-regulated genes in AD-astrocytes are associated mainly with synaptic signaling (i.e. *NRXN1* and *NRXN3*) and glutamate secretion (39). An independent study, showed that high *GFAP* astrocytes from AD, which lose homeostatic functions, also express pan-astrocytes and reactive markers such as *CD44*, *HSPB1*, *TNC* and *HSP90AA1* (40). Remarkably, some recent studies emphasize the gut-brain axis as an important player during the course of CNS disease that fine-tunes inflammation and neurodegeneration. In EAE, the deletion of aryl hydrocarbon receptor (AHR) in microglia, upregulated the expression of genes in astrocytes associated with inflammation and neurodegeneration (*Ccl2, Il1b* and *Nos2*) (41). A later study described a subset of LAMP1+ astrocytes limiting inflammation, driven by IFNγ produced by meningeal natural killer cells, which is modulated by the commensal flora in mice (42). Notably, Clark et al. use the RABID-seq technology to identify pro-inflammatory astrocytes connected to T cells that exhibited high TNFα signaling *via* NF-κB (8).

### Brain Tumors

In malignant brain tumors, knowledge related to astrocyte function and crosstalk to other components of the environment requires further investigation. Tumor-occupying astrocytes analyzed in three glioblastoma patients revealed similarities to highly proliferative astrocyte precursor cells from fetal brains (43). JAK/STAT pathway activation and *CD274* expression was present in RAs, in a set of *de-novo* and recurrent glioblastoma specimens, inducing immunological cold tumor environment (44). Notably, in the context of brain metastasis, a pro-metastatic program driven by STAT3 signaling in a subpopulation of RAs surrounding metastatic lesions promotes an immunosuppressive microenvironment, being an interesting target (45).

## Heterogeneity of Endothelial Cells Within the Brain Microenvironment

### Health

The lack of a molecular understanding of the constituent cell types of the brain vasculature could be solved by using single cell approaches. In murine models, single-cell transcriptomics distinguished different molecular signatures and phenotypic changes in endothelial and mural cells (46). Moreover, brain-specific endothelial transcripts have been identified, mainly cell surface transporters and intracellular enzymes (47).

### Brain Tumors

In a glioblastoma mouse model, single cell sequencing identified three separated clusters of brain endothelium with a distinct molecular signature, differentiating tumor associated vessels and tumor derived endothelial cells (detailed in **Table 1**) (48). Moreover, in human samples, heterogeneity was reported within pericytes and endothelial cells (49). These pioneer studies describe molecular inter and intra-heterogeneity within the primary brain tumor vasculature.

In human brain metastasis patients, clusters of endothelial cells have been identified using the marker CLDN5+, being in higher proportion in melanoma than in breast cancer brain metastasis (50).

## Therapeutic Strategies Exploiting the Heterogeneity Within the Brain Microenvironment

Uncovering functional and molecular diversity of glial and brain immune cells in preclinical models and patients affected by disease has a remarkable translational potential, including brain tumors. However, an important effort in the field is needed to validate the contribution of disease-associated alterations and cellular cross-talk between the various reactive states described.

### Brain Disorders

Ajami et al. proposed the surface marker CD49e found in peripheral monocytes, to be a therapeutic target in EAE since the treatment with anti-CD49e antibody significantly reduced disease severity (20). Interestingly, in the treatment of brain neurodegeneration, targeted immunotherapies may be used against B cell clusters responsible for disease-specific antibody production (51). Mapping the cross-talk between identified cell populations that shape the local microenvironment in brain disorders is key to uncover potential targets (8). Clark et al. have shown that in a EAE model, inactivating the interaction between *Sema4d-Plxnb2 or Ephb3-Efnb3* in microglia-astrocytes, respectively, ameliorates the disease (8). Interestingly, as a proof of concept in traumatic brain injury models (mTBI), Arneson et al. focused on the thyroid hormone pathway based on its differential expression across cell types in mTBI. Injecting T4 immediately after the damage improved cognitive deficits in a mouse model of concussive injury (52). In addition, specific gene expression programs related to endosome, plasma membrane, mitochondrion and autophagy have been shown to be relevant for the progression of neurodegeneration in humans, especially when enriched in neurons and microglia (53, 54). This finding emphasizes the emerging vulnerability of dysfunctional bioenergetics for brain disorders.

### Brain Tumors

Understanding the diversity within the microenvironment of clinically-relevant experimental models of brain tumors will help to identify altered pathways not essential for brain homeostasis. To illustrate this point, using single cell transcriptomics in the DNp53-PDGFB glioma model, Weng et al. have been able to identify the RNA-binding protein Zfp36l1 to be necessary for malignant oligodendrocyte-astrocyte lineage transition and glioma growth (55). In human primary and secondary tumors, candidate immunosuppressive molecules could be used to potentiate immunotherapy by designing customized strategies for brain tumors. For instance, by using single-cell gene expression Caruso et al. found TLR2 to be exclusively upregulated in glioma-associated microglia and CRTAM receptor in CD8+ T cells, confirming previous studies (56, 57) that have proposed these molecules as targets for adjuvant immunotherapies in glioma. Additionally, the same scRNAseq study defined ligand-receptor interactions between the microenvironment and cancer cells such as HBEGF-EGFR, MIF-CD74 and CD11B/CD18-CD90, that could be potential targets given their role in immunosuppression. FAB, identified mainly in endothelial cells and pericytes by scRNAseq, has been proposed as a potential antigen for (CAR)‐T cells therapy to target tumor cells and tumor associated vessels in glioblastoma (49).

Intrinsic properties of cancer cells could indirectly influence response to therapy by modulating the brain microenvironment. Whether the mutational status of cancer cells could influence the brain microenvironment in secondary brain tumors, as it does in primary brain tumors (58–62), is still unexplored. However changes in the immune infiltrate have been reported depending on the primary origin of brain metastases. For example, melanoma brain metastasis present higher frequencies of T cells than carcinoma brain metastasis, except for Tregs (28, 61). Notably, tumor location influences microenvironmental landscapes (63, 64). Meningiomas have higher TAM infiltration and less presence of T regs than gliomas (64), while in secondary brain tumors, scRNAseq revealed a distinct immune-suppressed T-cell microenvironment in leptomeningeal metastasis compared with brain metastasis derived from melanoma (63).

Furthermore, a deep knowledge of immune diversity induced by the presence of tumor cells is critical to predict immunotherapeutic outcomes since it might help to explain the different response to checkpoint inhibitors reported in primary and secondary brain tumors. For instance, Close et al. suggest that the presence of immune signatures with anti-tumor effector functions (i.e. granzyme B or IFN-γ) in a subset of patients with GBM will predispose to better benefit from combination immunotherapies (32). In addition, immune evasion signatures have been defined and novel targets, such as CDK4/6, have been proposed to overcome the resistance to immune checkpoint blockade in cancer metastatic to the brain (65, 66). scRNAseq of a melanoma brain metastasis patient found *PDCD4* to be expressed on CD8 Tcells, NK cells, B cells and mast cells, associated with cytotoxicity (Gzm expression), suggesting a role to potentiate immune response (67). Other very interesting tools are predictive studies of brain metastatic tumors to classify patients into potential good or bad responders to immunotherapy. This approach is able to determine specific molecules as targets for adjuvant immunotherapies according to the immune profile, which allows to narrow down candidates to specific biomarkers. For instance, the expression of CD74 in the microenvironment of brain metastases fulfilled the *in silico* criteria (68). Uncovering functional and dysfunctional CD8+ T cell activation states in brain tumors is key to establish more accurate immune signatures to stratify patients. Transposase-accessible chromatin sequencing (ATAC-seq) and RNA-seq could be applied to preclinical models of brain metastasis and human data to achieve this goal, as it has been done for hepatocarcinoma and melanoma (69). Other important aspect to consider is the reprogramming of the brain immune landscape by therapy. i.e. TMZ in primary tumors (60) and WBRT in brain metastasis (70).

Finally, due to the limited availability of brain tissue from patients, profiling the mutational landscape and evolutionary patterns of tumor and microenvironment using non-invasive biopsies, could be key to establish predictive biomarkers of therapeutic response. In this sense, pioneer studies using CSF as a relative non-invasive surrogate to be processed by single-cell techniques could help to define the heterogeneity of the immune microenvironment and its link to clinically meaningful correlations (34, 35). Analysis of this liquid biopsy in patients with positive and negative local responses to immunotherapy has started to be explored (61). Moreover, considering the important role of meningeal lymphatics in regulating brain tumor immunity, other plausible source of cancer-derived material is the regional lymphatic drainage (71), although in-depth analysis is needed to characterize the immune landscape in this liquid biopsy.

## Discussion

In conclusion, the data reviewed lays a firm foundation for considering vulnerabilities generated in the brain metastasis microenvironment relevant to predict and improve responses to immune based therapies that are effective only in a limited percentage of patients, especially when asymptomatic (72, 73). Overall, we consider a key aspect to embrace the emerging complexity and to dissect functionally relevant hubs within the local microenvironment, providing the avenues to transform the clinical management of brain metastasis patients within the years to come.

## Data Availability Statement

The original contributions presented in the study are included in the article/supplementary material. Further inquiries can be directed to the corresponding author.

## Author Contributions

LÁ-E, AP-A, MV, and NP conceptualized and wrote the manuscript. All authors contributed to the article and approved the submitted version.

## Funding

Research in the Brain Metastasis Group is supported by MINECO (SAF2017-89643-R) (MV), Fundació La Marató de TV3 (141) (MV), Fundación Ramón Areces (CIVP19S8163) (MV), Worldwide Cancer Research (19–0177) (MV), H2020-FETOPEN (828972) (MV), Cancer Research Institute (Clinic and Laboratory Integration Program CRI Award 2018 (54545) (MV), AECC (Coordinated Translational Groups 2017 (GCTRA16015SEOA) (MV), LAB AECC 2019 (LABAE19002VALI) (MV), ERC CoG (864759) (MV), La Caixa INPhINIT Fellowship (LCF/BQ/DI19/11730044) (AP-A), MINECO-Severo Ochoa PhD Fellowship (BES-2017-081995) (LA-E), AECC Postdoctoral Fellowship (POSTD19016PRIE) (NP). MV is an EMBO YIP investigator (4053).

## Conflict of Interest

The authors declare that the research was conducted in the absence of any commercial or financial relationships that could be construed as a potential conflict of interest.

## Publisher’s Note

All claims expressed in this article are solely those of the authors and do not necessarily represent those of their affiliated organizations, or those of the publisher, the editors and the reviewers. Any product that may be evaluated in this article, or claim that may be made by its manufacturer, is not guaranteed or endorsed by the publisher.
